# Pharmacotherapy of Liver Fibrosis and Hepatitis: Recent Advances

**DOI:** 10.3390/ph17121724

**Published:** 2024-12-20

**Authors:** Liangtao Zhao, Haolan Tang, Zhangjun Cheng

**Affiliations:** 1 Hepato-Pancreato-Biliary Center, Zhongda Hospital, School of Medicine, Southeast University, Nanjing 210009, China; ahzlt2008@163.com; 2 School of Medicine, Southeast University, Nanjing 210009, China; zdyytanghaolan@163.com

**Keywords:** liver fibrosis, hepatitis, hepatic stellate cells, molecular mechanisms, antifibrotic agents, chronic inflammation, drug-induced liver injury, autoimmune liver disease, biomarkers, therapeutic targets

## Abstract

Liver fibrosis is a progressive scarring process primarily caused by chronic inflammation and injury, often closely associated with viral hepatitis, alcoholic liver disease, metabolic dysfunction-associated steatotic liver disease (MASLD), drug-induced liver injury, and autoimmune liver disease (AILD). Currently, there are very few clinical antifibrotic drugs available, and effective targeted therapy is lacking. Recently, emerging antifibrotic drugs and immunomodulators have shown promising results in animal studies, and some have entered clinical research phases. This review aims to systematically review the molecular mechanisms underlying liver fibrosis, focusing on advancements in drug treatments for hepatic fibrosis. Furthermore, since liver fibrosis is a progression or endpoint of many diseases, it is crucial to address the etiological treatment and secondary prevention for liver fibrosis. We will also review the pharmacological treatments available for common hepatitis leading to liver fibrosis.

## 1. Introduction

Liver fibrosis (LF) is a progressive condition characterized by the excessive accumulation of extracellular matrix (ECM) components, leading to liver scarring and the potential progression to cirrhosis. Epidemiological studies indicate that this condition is a significant global health burden, with estimates suggesting that approximately one in five adults exhibits some degree of liver fibrosis [1]. The clinical symptoms of liver fibrosis vary and can include fatigue, weakness, decreased appetite, nausea, vomiting, indigestion, and upper abdominal pain, often accompanied by bleeding. Currently, clinical diagnosis primarily relies on liver biopsies, biochemical tests, and imaging methods [2].

The consequences of liver fibrosis are profound due to its potential progression to cirrhosis, liver failure, and hepatocellular carcinoma, leading to significant morbidity and mortality [3]. Additionally, liver fibrosis can have adverse effects on systemic health, contributing to complications such as portal hypertension and extrahepatic manifestations [4].

Liver fibrosis results from two main types of chronic liver injuries: hepatotoxic injury and cholestatic injury. Hepatotoxic injury is typically caused by chronic damage to hepatocytes due to factors such as hepatitis B (HBV) and/or hepatitis C (HCV) infections, alcohol consumption, or non-alcoholic steatohepatitis (NASH), resulting from metabolic syndrome [5]. In contrast, cholestatic injury occurs due to the obstruction of bile flow, which occurs in primary (and secondary) biliary cholangitis (PBC), primary sclerosing cholangitis (PSC), and biliary atresia. These injuries trigger a series of pathological changes in the liver. The pathophysiological mechanisms underlying liver fibrosis involve complex interactions among hepatocytes, hepatic stellate cells, and inflammatory mediators [6]. Notably, the activation of hepatic stellate cells plays a central role in the fibrogenic response, as these cells transdifferentiate into myofibroblast-like cells that produce collagen (primarily cross-linked types I and III) and other matrix proteins in response to liver injury [7,8].

Organ fibrosis is reported to account for approximately 45% of all-cause mortality worldwide [9]. Despite extensive efforts to develop new antifibrotic drugs, drug discovery has not kept pace with clinical demand. At present, surgical intervention remains the only effective therapeutic option for patients with advanced stages of fibrosis [10]. To date, the FDA has approved pirfenidone and nintedanib for treating pulmonary fibrotic diseases, which raises hopes for the exploration of drug treatments for liver fibrosis. A variety of targeted therapeutic agents, including selective antagonists and agonists, are currently under clinical investigation for the treatment of liver fibrosis. These compounds encompass trimetazidine, a metabolic modulator; statins, which are lipid-lowering agents with pleiotropic effects; elafibranor, a dual peroxisome proliferator-activated receptor (PPAR)-alpha and -delta agonist; PRI-724, a novel synthetic compound with antifibrotic properties; and heat shock proteins (HSPs), which are molecular chaperones involved in cellular stress responses. Each of these therapeutic candidates represents a unique mechanism of action and potential clinical application in the management of liver fibrosis, highlighting the diversity of approaches within the field [11]. Ongoing investigation into the mechanisms of liver fibrosis and the development of innovative therapeutic strategies will be essential for improving the management of this challenging condition.

Liver fibrosis generally occurs due to factors, such as alcohol abuse, obesity, viral infections, autoimmune diseases, or the accumulation of certain drugs and toxins in the liver. The treatment of fibrosis is complicated by the fact that its underlying causes are often not simultaneously addressed, underscoring the importance of targeting both the fibrosis and its etiology [12]. This highlights the necessity of understanding the molecular mechanisms driving hepatic inflammation to develop therapies aimed at preventing severe complications and promoting the regression of liver disease.

This review summarizes the latest advancements in hepatic fibrosis, with a focus on the molecular mechanisms and potential therapeutic targets. It also highlights recent progress in drug therapies for various types of hepatitis that lead to liver fibrosis.

## 2. Mechanisms of Liver Fibrosis Formation

**Figure 1:**
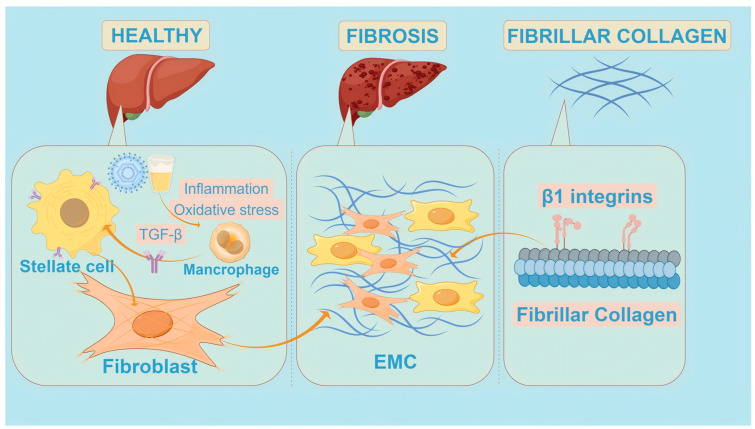
Illustration of the cause of liver fibrosis (from Figdraw, www.figdraw.com).

Liver fibrosis is a progressive condition characterized by the excessive accumulation of extracellular matrix (ECM) components in the liver, which can potentially lead to cirrhosis and liver failure. As shown in Figure 1, while collagens constitute the most abundant ECM components and increase up to tenfold in cirrhosis, numerous other ECM molecules serve as indicators or therapeutic targets for fibrosis treatment [13]. Beyond collagens, various ECM molecules regulate the availability of fibrogenic or fibrolytic mediators and signal to ECM receptors on cells involved in fibrosis progression or reversal.

The initial phase of liver fibrosis often results from liver injury caused by chronic conditions, including the presence of toxins, viral infections, cholestasis, hypoxia, and harmful metabolites [14]. For example, insulin resistance, commonly seen in non-alcoholic steatohepatitis (NASH), leads to the accumulation of free fatty acids and toxic lipids, excess oxidative stress, mitochondrial dysfunction, and hepatocyte lipoapoptosis. Prolonged exposure to these injuries triggers progressive fibrogenesis through the induction of profibrogenic cytokines and growth factors that activate the downstream effectors of fibrosis (Figure 1).

These injuries prompt the release of pro-inflammatory cytokines and chemokines that recruit immune cells. Among these, macrophages or Kupffer cells play a pivotal role in mediating inflammation and promoting fibrogenesis through the secretion of cytokines such as TGF-β, which stimulate HSC activation [15,16,17,18] (Figure 1). Upon activation, HSCs transform into myofibroblast-like cells capable of producing collagen and other ECM components, contributing to the fibrotic matrix and disrupting the normal liver architecture [19]. In advanced fibrosis, HSCs and myofibroblasts can also be activated by a stiff ECM, predominantly composed of fibrillar collagen, which sends signals to contract and promote ECM synthesis, primarily via β1 integrins [20].

**Figure 2:**
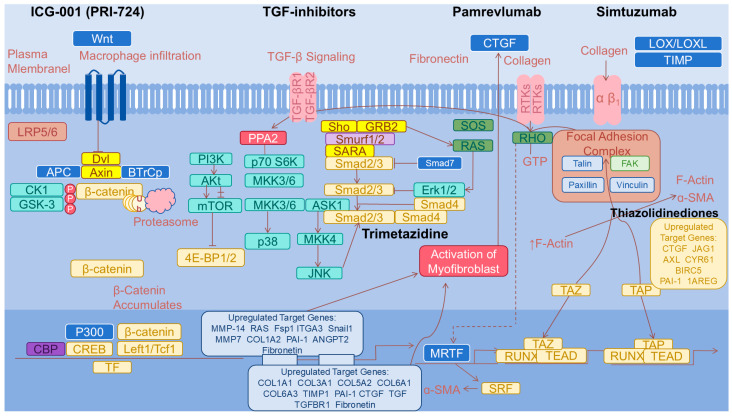
Pathway diagram of liver fibrosis mechanism (from Figdraw, www.figdraw.com).

The liver microenvironment plays an important role in the formation of liver fibrosis. Factors such as the presence of inflammatory cells, particularly macrophages, and the ECM’s composition can also significantly influence HSC activation and proliferation [21]. During liver injury, activated hepatic stellate cells (HSCs), macrophages, endothelial cells, and hepatocytes produce the platelet-derived growth factor (PDGF), which plays a key role in liver fibrosis. By binding to its receptor (PDGFR), PDGF activates downstream signaling pathways (such as the PI3K/Akt and MAPK pathways), promoting the activation and proliferation of hepatic stellate cells and enhancing collagen synthesis, thereby exacerbating liver fibrosis. TNF-α produced by immune cells also plays multiple roles in liver fibrosis, including promoting inflammatory responses, activating hepatic stellate cells, and enhancing collagen synthesis. The complex regulatory network of liver fibrosis also includes IL-1β, IL-6, IL-10, the Wnt/β-catenin pathway, the Notch pathway, the Hippo pathway, and the regulation of autophagy (Figure 2).

Additionally, dysregulation of signaling pathways, particularly the JAK/STAT3 pathway, is a significant contributor to liver fibrosis. Inhibitors targeting these pathways have demonstrated promise in preclinical models by reducing HSC activation and collagen deposition, highlighting potential therapeutic targets [22,23]. The expression of certain genes, including HOXB13, has been found to correlate with inflammatory activity associated with liver fibrosis, suggesting that genetic factors may influence the fibrogenic response [24] (Figure 2).

In summary, the mechanisms underlying hepatic fibrosis formation are complex, involving numerous cellular interactions and signaling pathways. A deeper understanding of these mechanisms is pivotal for developing effective therapeutic strategies to prevent or reverse liver fibrosis, especially in patients with chronic liver disease. Further research is needed to unravel the intricate networks involved in fibrogenesis and to explore potential interventions that effectively target these pathways.

## 3. Diagnostic Markers of Liver Fibrosis

The early diagnosis of liver fibrosis is essential for preventing its progression to cirrhosis and liver failure. Various diagnostic markers have been identified to assess the degree of liver fibrosis with the use of non-invasive blood tests and imaging techniques [25,26]. Among these, serum biomarkers such as hyaluronic acid, collagen type IV, and tissue inhibitors of metalloproteinases (TIMPs) have garnered attention due to their association with liver histology. For example, elevated levels of hyaluronic acid are linked to hepatic inflammation and fibrosis, suggesting its potential as a marker for liver disease progression [21]. Moreover, the role of hepatic macrophages in fibrosis development indicates that their activity could also serve as a marker for liver fibrosis [17].

Recent studies have investigated the utility of combining several biomarkers to enhance diagnostic accuracy. The integration of non-invasive tests, such as the Fibrosis-4 (FIB-4) index and the aspartate aminotransferase-to-platelet ratio index (APRI), has shown promise in stratifying patients based on the severity of fibrosis [17]. Additionally, advancements in molecular diagnostics, including the assessment of circulating microRNAs and their expression profiles, have opened new avenues for non-invasive liver fibrosis detection [19]. Ongoing research aims to refine these biomarkers and validate their clinical application across diverse populations, thereby improving early diagnosis and management strategies for patients at risk of liver fibrosis [26,27,28].

In conclusion, the landscape of diagnostic markers for liver fibrosis is evolving, with a focus on identifying reliable, non-invasive methods that accurately reflect the underlying liver pathology. As our understanding of liver fibrosis deepens, the integration of novel biomarkers into clinical practice is anticipated to enhance patient outcomes and facilitate timely interventions.

## 4. Drug Treatment of Liver Fibrosis

Hepatic fibrosis, a pathological condition of the liver characterized by liver cell injury, regeneration, extracellular matrix accumulation, and hepatic stellate cell activation, can result from various factors. It represents the final stage of most severe hepatic diseases and is a leading cause of mortality in patients [29]. The cause of liver fibrosis is complex, and its treatment is difficult. Currently, few drugs are approved for the treatment of liver fibrosis. Ursodeoxycholic acid (C_24_H_40_O_4_, UDCA) is approved for the treatment of primary biliary cirrhosis and primary sclerotic cholangitis, but patients with other conditions may only partially respond to UDCA.

Obeticholic acid is approved for the treatment of primary biliary cholangitis [30], but it causes significant adverse reactions, such as cardiovascular risk, bile stasis, and itching. Patients with liver fibrosis urgently need effective, specific drugs. With a growing understanding of the mechanisms of liver fibrosis, drug research is increasingly targeting the inhibition of hepatic stellate cell activation, the alleviation of liver inflammation, and the regulation of the liver microenvironment. Colchicine, ligands of peroxisome proliferator-activated receptor (PPAR)-γ, renin–angiotensin system inhibitors, herbal remedies, and pirfenidone are among the drug candidates that have shown potential in inhibiting collagen synthesis and hepatic stellate cell activation for treating liver fibrosis [5]. Additionally, antioxidants, such as phosphatidylcholine, vitamin E, S-adenosyl L-methionine, and silymarin, have been evaluated for their protective effects against oxidative stress that induces liver fibrosis and injury [31].

### 4.1. Direct Targeting of Fibrotic Components

Several research studies have focused on targeting the specific components of the liver to treat fibrosis. Some examples are described below.

Simtuzumab, an inhibitor of the LOXL2 (Lysyl Oxidase-Like 2) enzyme, has been studied in clinical trials. LOXL2 is an important enzyme in the lysyl oxidase family. It primarily catalyzes the oxidation of lysine within the body and is involved in the remodeling of the extracellular matrix, including the cross-linking of collagen and elastin, thus playing a crucial role in the structural stability and elasticity of tissues. Simtuzumab is a humanized monoclonal antibody targeting LOXL, but it failed to improve liver fibrosis, leading to its termination in phase II clinical trials [32]. Chen et al. suggested that the poor efficacy of simtuzumab may be due to the low effectiveness of the antibody, but this does not necessarily indicate that LOXL2 is an unsuitable target for the treatment of liver fibrosis [33] (Table 1).

Pamrevlumab (FG-3019) is a fully human recombinant monoclonal antibody targeting connective tissue growth factor (CTGF) [34]. CTGF is a cytokine in the CCN family of proteins. CTGF plays a significant role in the process of fibrosis, promoting the proliferation of fibroblasts and the synthesis of collagen, which can lead to tissue fibrosis, as is particularly evident in diseases such as liver fibrosis, pulmonary fibrosis, and renal fibrosis. Due to its weaker efficacy in improving liver fibrosis in trials compared to entecavir, pamrevlumab’s development is currently paused in phase II clinical trials.

BMS-986263: Heat shock protein 47 (HSP47) activity is characterized by the increased expression of type I and type III collagen, a hallmark of liver fibrosis [35]. HSP47 acts as a molecular chaperone, binding to triple-helical procollagen in the endoplasmic reticulum to prevent the improper unfolding or aggregation of collagen [36]. Research indicates that the mRNA and protein levels of HSP47 are significantly elevated in fibrotic tissues, suggesting its role in collagen accumulation and the progression of fibrosis [37]. BMS-986263 is a small interfering RNA (siRNA) targeting HSP47, delivered in vitamin-A-conjugated lipid nanoparticles (LNPs). These LNPs contain retinol, allowing them to bind to retinol-binding protein expressed on hepatic stellate cells (HSCs), facilitating the targeted uptake of BMS-986263 by these cells. The HSP47 siRNA recruits the RNA-induced silencing complex, which degrades HSP47 mRNA and prevents the translation of the HSP47 protein. Consequently, the inhibition of HSP47 mRNA by BMS-986263 may reduce or reverse liver fibrosis by disrupting collagen formation and promoting the apoptosis of stellate cells. BMS-986263 has been tested in clinical trials involving healthy volunteers and patients with advanced liver fibrosis (stage F3 or F4) secondary to non-alcoholic steatohepatitis (NASH) or chronic hepatitis C (HCV). The treatment regimen involves administering BMS-986263 once a week for 12 weeks and has demonstrated good tolerability in patients with advanced fibrosis, with liver biopsies indicating improvements in tissue pathology (Table 1).

Belapectin (GR-MD-02): Galectin-3 (Gal-3) is a key protein involved in fibrosis across various organs, and its inhibition may help prevent liver fibrosis [38]. GR-MD-02 is a Gal-3 inhibitor that showed good safety in early trials, but its efficacy in reducing fibrosis in patients with chronic liver diseases remains to be fully established [39]. Although it showed some benefits in specific patient subgroups, larger and more targeted studies are needed to better understand its therapeutic potential in liver fibrosis (Table 1).

### 4.2. Inhibition of Hepatic Stellate Cell Activation

Hepatic stellate cells (HSCs) play a crucial role in liver fibrosis, which is characterized by excessive ECM deposition. Their activation is a key event in the pathogenesis of liver fibrosis, during which quiescent HSCs transform into myofibroblast-like cells that proliferate and produce collagen. Therefore, targeting HSC activation has emerged as a therapeutic strategy to mitigate liver fibrosis. Various pharmacological agents have been investigated for their efficacy in inhibiting HSC activation (Table 1).

Thiazolidinediones: PPARs (peroxisome proliferator-activated receptors) are ligand-activated receptors belonging to the nuclear hormone receptor family, which includes three subtypes: PPARα, PPARβ/δ, and PPARγ. PPARγ can inhibit the activation and proliferation of quiescent hepatic stellate cells (HSCs) and induce cell cycle arrest and apoptosis, thereby restraining liver fibrosis [40]. Increasing the expression of exogenous PPARγ can suppress the synthesis of type I collagen, as well as the expression of α-SMA and hydroxyproline in HSCs, reducing the degree of liver fibrosis. Therefore, PPAR-γ ligands and agonists can prevent the occurrence of liver fibrosis. Thiazolidinediones, a class of PPARγ ligands, including drugs such as Rosiglitazone and Pioglitazone, are widely used in the treatment of diabetes and have been proven to effectively reduce the degree of liver fibrosis in animal studies [41]. Unfortunately, thiazolidinediones have not demonstrated significant antifibrotic effects in clinical trials. In a randomized, double-blind clinical trial of Rosiglitazone, subjects with non-alcoholic liver cirrhosis were either treated with Rosiglitazone or a placebo for one year. Although the Rosiglitazone group showed improvements in the levels of steatosis and transaminases compared to the placebo group, there was no significant improvement in liver fibrosis or other histological conditions. This is partly because thiazolidinediones can increase plasma adiponectin levels by 2.3-times. While the increase in adiponectin may improve insulin resistance, glucose tolerance, glucose clearance, fatty degeneration, and necrotizing inflammation, it may concurrently elevate liver fibrosis levels [42]. Therefore, in practical treatment, combining other medications with thiazolidinediones may be necessary (Table 1).

ICG-001 (PRI-724): Selective CBP-β-catenin antagonists, such as ICG-001 (PRI-724), target the Wnt/β-catenin signaling pathway, which plays a significant role in fibrosis in various organs, including the liver [43]. This pathway interacts with others, such as TGF-β and NF-κB, to activate hepatic stellate cells and promote their transformation into fibroblasts [44]. ICG-001 specifically inhibits CBP-mediated transcription, which is essential for cellular proliferation, while allowing for the modulation of differentiation through p300 [45]. Clinical trials have indicated that ICG-001 is safe and tolerable in patients with HCV-related cirrhosis, with nausea and fatigue as the most frequently reported side effects [46] (Table 1).

TGF-β inhibitors have good prospects in the treatment of liver fibrosis, as they can slow the progression of fibrosis by inhibiting the activation of hepatic stellate cells and collagen synthesis. For example, GC1008, SD-208, and IPW-5371 have all demonstrated effects on liver fibrosis or other fibrotic disorders in animal experiments [47,48,49]. However, since TGF-β also plays important roles in many physiological processes, treatment targeting this factor needs to carefully consider its potential side effects and systemic impacts. Further research and clinical trials are still essential (Table 1).

PDGF inhibitors: PDGF (platelet-derived growth factor) plays an important role in the development of liver fibrosis by promoting the proliferation and activation of fibroblasts, thereby accelerating the fibrotic process in the liver. PDGF inhibitors have shown potential in the treatment of liver fibrosis, as they may effectively slow fibrotic progression by reducing the proliferation and activation of fibroblasts. Imatinib and Sorafenib, both tyrosine kinase inhibitors, can also inhibit PDGF and have demonstrated good antifibrotic effects in animal experiments [50,51]. Nintedanib and Crenolanib also have potential applications in the treatment of fibrosis in other organs, and they may also play a role in the treatment of liver fibrosis in the future [52,53]. However, further research and clinical trials are still necessary to determine their safety, efficacy, and optimal treatment regimens. Research in this field also brings new therapeutic hope to patients with chronic liver disease (Table 1).

It is encouraging that other targets that act on hepatic stellate cells are expected to bring hope for the treatment of liver fibrosis. For example, novel antifibrotic agents such as trimetazidine have been shown to inhibit HSC proliferation and block the TGF-β/Smad signaling pathway, demonstrating promising results in both in vitro and in vivo models of liver fibrosis [16]. The rSjp40 agent is reported to induce the nuclear translocation of YB1, which subsequently stimulates the BMP-7/Smad1/5/8 pathway and inhibits activated HSCs [54]. The Rab [55] protein has been identified as a promoter of HSC activation by accelerating the endocytosis of the TGF-β receptor II complex, revealing a novel mechanism of HSC activation [56]. Furthermore, the involvement of exosomes in modulating HSC function presents an innovative therapeutic avenue. Advances in understanding the regulatory roles of exosomal components could pave the way for new drug delivery systems targeting HSCs [57]. The modulation of inflammatory pathways, particularly those mediated by Toll-like receptors, also suggests that anti-inflammatory strategies may complement therapies that directly target HSCs [58]. The development of nanoparticle-based drug delivery systems offers a new approach to enhancing drug delivery specifically to fibrotic liver tissues, increasing therapeutic efficacy while minimizing systemic side effects [55]. Researchers have developed a CREKA-modified polyethylene glycol liposome for the targeted delivery of sorafenib, targeting the high expression of fibronectin on HSCs. This system effectively targets HSCs both in vitro and in vivo, significantly alleviating CCl(4)-induced liver fibrosis, abnormal angiogenesis, and inflammation [59]. Another team has developed a silica cross-linked micelle (SCLM)-based nano-system for the diagnosis and treatment of liver fibrosis. These SCLMs are first modified with the peptide CTCE9908 (CT-SCLMs), which can actively target the overexpression of CXCR4 in activated hepatic stellate cells (HSCs), enabling early diagnosis. For therapeutic purposes, the nano-system also co-encapsulates two antifibrotic drugs, silibinin and sorafenib, significantly alleviating liver injury. Experimental results indicate that symptoms associated with liver fibrosis, such as collagen deposition, hydroxyproline expression, and elevated serological markers, show significant improvement [60]. Innovations in targeted drug delivery to HSCs highlight a promising method for preventing fibrosis formation and progression, especially concerning hepatocellular carcinoma [61] (Table 1).

Recent research has also explored the potential of natural compounds in inhibiting HSC activation. For example, compounds such as curdione and Schisandrin C have shown synergistic effects in reversing hepatic fibrosis by modulating the TGF-β pathway and inhibiting oxidative stress, demonstrating their therapeutic potential [62]. Moreover, hyaluronic acid-modified extracellular vesicles targeting HSCs are a promising strategy for attenuating liver fibrosis, indicating a shift toward more targeted and less invasive therapeutic approaches [63].

### 4.3. Inhibition of Hepatic Inflammatory Response

The process of liver fibrosis is closely linked to chronic inflammation, which is pivotal in the progression of liver diseases. Inhibiting inflammatory responses is vital for developing effective antifibrotic therapies. Modulating inflammatory pathways not only aids in preventing fibrosis but also promotes the resolution of existing fibrotic tissue. Integrating anti-inflammatory agents into fibrotic disease management could enhance the efficacy of existing treatments and improve patient outcomes [64]. Current investigations focus on established medications with potential antifibrotic properties, leveraging their known safety profiles to expedite clinical application. The exploration of repurposed FDA-approved drugs has also gained traction as a significant strategy in treating liver fibrosis (Table 1).

Apoptosis signal-regulating kinase 1 (ASK1) inhibitors, such as Gilead’s selonsertib (C_24_H_24_FN_7_O, GS4977), are important in managing liver cell damage, inflammation, and fibrosis. ASK1 activation leads to pro-inflammatory and fibrotic changes through the MAPK pathway, and inhibiting ASK1 can counteract these effects. Phase II trials of selonsertib demonstrated its potential to reduce liver fibrosis in NASH patients. However, in two ongoing phase III trials, selonsertib did not achieve the primary endpoint of improving fibrosis in patients with more advanced stages (F3 and F4), suggesting that its effectiveness may be limited to earlier stages of liver fibrosis (F1–F3) [65] (Table 1).

BRD4 (Bromodomain-Containing Protein 4) inhibitors are a class of compounds that target BRD4, an epigenetic regulator capable of recognizing and binding to acetylated lysines. Recent studies have shown that BRD4 plays an important role in organ fibrosis (e.g., liver fibrosis and lung fibrosis), and BRD4 inhibitors are expected to alleviate these conditions [66]. Although approximately 1700 BRD4 inhibitors have been developed, only a few have been evaluated in clinical trials. Some, such as JQ1, I-BET151 (GSK1210151A), and Compound 38, have been studied and shown to improve liver fibrosis, demonstrating strong preclinical and clinical potential [67,68,69]. However, the use of BRD4 inhibitors warrants caution due to their effects on normal cells, as well as potential teratogenicity and adverse reactions (Table 1).

Angiotensin II plays an important role in the development of liver fibrosis, primarily by promoting oxidative stress, stimulating the production of growth factors (e.g., TGF-β1), and enhancing the activation of hepatic stellate cells, which, in turn, leads to collagen deposition and the progression of liver fibrosis [70]. Additionally, the activation of AT1R is a crucial regulatory mechanism in this process. Therefore, drugs targeting Angiotensin II and its receptors (such as ACE inhibitors and ARBs) are considered potential therapeutic strategies to slow the progression of liver fibrosis. Clinical trials involving candesartan (an ARB) have demonstrated that, when combined with UDCA, it improves histological outcomes in patients with compensated alcohol-related liver fibrosis. For patients with hypertension and hepatitis C, candesartan also proved more effective in reducing liver fibrosis than other antihypertensive agents [71]. However, the use of sartans in non-cardiovascular contexts may face challenges due to dose-dependent hypotension, particularly in advanced liver diseases [72]. As a result, combining ACEIs and ARBs is considered a potentially effective therapeutic strategy for patients with hypertension and liver fibrosis (Table 1).

FXR is a ligand-activated nuclear hormone receptor and a key regulatory factor in the synthesis, conjugation, and excretion of bile acids. FXR is highly expressed in the liver, gallbladder, intestines, and kidneys. Bile acids or FXR agonists activate FXR in the intestines, which reduces bile acid synthesis, enhances bile acid excretion, and decreases hepatic uptake of bile acids, thereby alleviating cholestasis and improving liver fibrosis [73]. Several FXR agonists are currently being tested in clinical trials, including OCA, cilofexor (GS-9674), and tropifexor (LJN452). In a phase III clinical trial, OCA was administered to subjects with primary biliary cholangitis (PBC) who had an inadequate response to or could not tolerate UDCA, resulting in significant improvements in the degree of liver fibrosis [74]. In May 2016, the US FDA approved OCA for use in combination with UDCA for PBC patients who had an inadequate response to UDCA monotherapy or as a monotherapy for PBC patients who could not tolerate UDCA. In non-cirrhotic and non-NASH patients, OCA can improve the degree of liver fibrosis. In a phase III clinical trial, subjects with NAFLD or fibrosis stages F1 to F3 who were treated with OCA for 18 months showed a reduction in liver fibrosis stages and an improvement in liver fibrosis [75]. In a phase II clinical trial, cilofexor (GS-9674) led to reductions in liver stiffness and the degree of liver fibrosis in NASH patients [76]. In a phase II clinical trial, cilofexor (GS-9674) was able to lower bile acids, ALP, gamma-glutamyl transferase, alanine aminotransferase (ALT), and AST in patients with primary sclerosing cholangitis (PSC), improving cholestasis and liver fibrosis in these patients [77]. Tropifexor (LJN452) is a non-bile-acid FXR agonist currently undergoing phase I clinical trials, with good tolerance reported [77] (Table 1).

Silymarin is a natural compound derived from the milk thistle plant (Silybum marianum) and consists primarily of active components such as silybin, silydianin, and silychristin. It is known for its various health benefits, particularly for liver health. Silymarin exerts its effects through multiple mechanisms, including strong antioxidant activity that protects liver cells from oxidative damage, anti-inflammatory properties that reduce hepatic inflammation, the promotion of liver cell regeneration to aid in tissue repair, and antifibrotic effects that inhibit the activation of hepatic stellate cells, highlighting its potential in treating liver fibrosis [78]. However, more extensive clinical trials are needed to confirm its effectiveness and safety for broader use in medical practice (Table 1).

Phyllanthus urinaria L. is derived from a plant in the Euphorbiaceae family. Research shows that Phyllanthus urinaria L. mainly contains flavonoids, lignans, tannins, phenolic acids, and terpenoids, which can exert antiviral hepatitis effects by reducing surface antigen secretion and interfering with DNA polymerase synthesis. It can also combat liver fibrosis/cirrhosis by decreasing the activity of hepatic stellate cells, inflammation, and oxidative stress [79]. Although many studies support its medicinal properties, further research is needed to fully understand its mechanisms of action and potential therapeutic applications (Table 1).

Other phytochemicals have emerged as promising candidates in combating liver fibrosis due to their anti-inflammatory properties. For instance, the antifibrotic properties of isoliquiritigenin, which inhibits HSC activation, are linked to its anti-inflammatory effects [80]. The combination of pentoxifylline and vitamin E has shown potential in inflammatory bowel disease by inhibiting intestinal fibrosis, highlighting the importance of addressing inflammation in fibrotic conditions [81]. Additionally, saffron has proven effective in reducing liver fibrosis in animal models by inhibiting the JAK/STAT3 signaling pathway, which is often activated in inflammatory responses [23].

With the advent of nanotechnology in medicine, nanoparticle-based drug delivery systems have emerged as a promising strategy to address the limitations of traditional drug delivery. These systems can enhance therapeutic efficacy while minimizing side effects by improving drug solubility, stability, circulation time, and targeting capabilities [82,83]. Nanoparticles can be engineered to specifically target liver cell types, further improving treatment effectiveness [84]. One study investigated the delivery of a mitogen-activated protein kinase (MAPK) inhibitor and sorafenib using carbon tetrachloride nanoparticles, which demonstrated antifibrotic effects by preventing extracellular-signal-regulated kinase (ERK) activation. Another report demonstrated that siCo11α1 and silibinin-filled vitamin A-coated micelles are biocompatible and safe for fibrous collagen I suppression, offering a unique clinical approach to treating hepatic fibrosis [85]. Additionally, other antitumor agents, such as GNS561, an agent with both antifibrotic and pro-fibrolytic effects, have been shown to inhibit transforming growth factor-beta 1 (TGF-β1)—a key cytokine involved in both fibrosis and inflammation—thereby exhibiting therapeutic potential [86]. In addition to pharmacotherapy, the interplay between the liver microenvironment and the progression of fibrosis is critical. Hepatic macrophages have been identified as crucial players in the fibrogenic process. Targeting these immune cells may provide dual benefits, reducing inflammation while also inhibiting fibrosis [87].

In conclusion, the pharmacological landscape for liver fibrosis treatment is rapidly evolving, with a focus on both novel and repurposed agents. The integration of advanced drug delivery systems holds promise for developing effective therapies capable of reversing liver fibrosis and improving patient outcomes, in addition to deepening our understanding of the cellular and molecular mechanisms involved. Continued research and clinical trials will be essential in validating these approaches and establishing standardized treatment protocols for effective liver fibrosis management.

**Table 1:** Drugs with different mechanisms of action and the latest research.

Mechanisms	Drug Name	Target	Latest Research	References
Direct Targeting of Fibrotic Components	Simtuzumab (C_6558_H_10134_N_1736_O_2037_S_50_)	LOXL/LOXL2	NCT02466516	[88,89,90]
	Pamrevlumab (FG-3019)	CTGF	NL-OMON54230	
	BMS-986263	HSP47 mRNA	NCT06646276	
	GR-MD-02 (belapectin)	Gal-3	ChiCTR2400091049	
Inhibition of Hepatic Stellate Cell Activation	Thiazolidinediones (C_3_H_7_NS)	PPARs ^a^	CTRI/2024/10/075038	[91,92,93,94]
	ICG-001 (PRI-724)	Wnt-β-catenin	NCT06647901	
	TGF-β inhibitors *	TGF-β	-	
	PDGF inhibitors *	PDGF	-	
Inhibition of Hepatic Inflammatory Response	ASK1 inhibitors *	MAPK pathway	-	[95,96,97,98]
	BRD4 inhibitors *	Acetylated lysines	-	
	Candesartan (C_24_H_20_N_6_O_3_)	Angiotensin II, AT1R	NCT06646354	
	OCA(C_26_H_44_O_4_), cilofexor (C_28_H_22_Cl_3_N_3_O_5_, GS-9674), tropifexor (C_29_H_25_F_4_N_3_O_5_S, LJN452)	FXR ^c^	NCT04971785	
	Silymarin(C_25_H_22_O_10_) *	-	IRCT20240720062482N1	
	*Phyllanthus urinaria* L *	-	-	

^a^, ^c^, * There are no relevant clinical studies.

## 5. Various Types of Liver Inflammation Causing Liver Fibrosis

### 5.1. MASLD/MASH and Liver Fibrosis

Metabolic dysfunction-associated steatotic liver disease (MASLD) and its more severe form, metabolic dysfunction-associated steatohepatitis (MASH)—formerly known as non-alcoholic fatty liver disease/non-alcoholic steatohepatitis (NAFLD/NASH)—encompass a spectrum of liver diseases characterized by fat accumulation in the liver due to metabolic dysfunction, often associated with obesity, type 2 diabetes, and dyslipidemia. Recent studies have indicated that the prevalence of MASLD in the general population ranges from 25% to 45%, with particularly elevated rates in specific demographics, including individuals with metabolic disorders and certain ethnic groups [99].

MASH is not simply characterized by a fatty liver; its pathophysiology includes hepatocyte injury, inflammation, and the progression of fibrosis. Therefore, early diagnosis of and intervention in MASH are crucial for preventing and treating liver fibrosis.

The pathophysiology of MASLD is complex, involving a multifaceted interplay of insulin resistance, dysregulated lipid metabolism, and inflammatory processes. Insulin resistance leads to increased lipolysis in adipose tissue, resulting in elevated free fatty acids in the liver, which promote steatosis. Concurrently, the liver’s capacity to export lipids is impaired, further increasing fat accumulation. This accumulation triggers inflammatory responses, activating hepatic macrophages and releasing pro-inflammatory cytokines such as TNF-α and IL-6, which exacerbate liver injury and promote fibrosis through the activation of hepatic stellate cells (HSCs) [100,101].

Recent experimental studies have identified a significant link between inflammasome activation and liver pathology in MASH models. For instance, mice lacking the NLRP3 gene and fed a high-fat diet exhibited worsened liver steatosis, increased macrophage infiltration, and heightened liver injury [102]. This was associated with intensified adipose tissue inflammation, enhanced insulin resistance, and alterations in gut microbiota that led to increased bacterial translocation [100,101,102,103]. Similarly, NLRP3-, CASP1-, and ASC-deficient mice consuming a methionine-/choline-deficient diet (MCDD) showed elevated serum transaminases and exacerbated liver steatosis compared to wild-type mice [104]. The liver inflammation observed in these inflammasome-deficient models appears to be driven primarily by intestinal dysbiosis, affecting local microbiota and reflecting the absence of inflammasome activity in intestinal epithelial cells. This dysbiosis can lead to the translocation of pathogen-associated molecular patterns (PAMPs) into the portal circulation, activating TLR4 and TLR9 in the liver and resulting in the release of TNF-α, contributing to hepatotoxicity from altered microbiota. The increased liver steatosis in inflammasome knockout models correlates with the upregulation of genes associated with lipid uptake and storage, alongside decreased antioxidant responses.

Innovative animal models with the liver-cell-specific knockout of the NLRP3 gene provide insights into NLRP3-mediated cell communication. Research indicates that myeloid cells primarily mediate NLRP3 activation in the liver, contributing to neutrophil infiltration and influencing the pro-fibrotic phenotype of HSCs [105]. Furthermore, the role of IL-18 in activating HSCs and fostering liver fibrosis has been demonstrated, as the antagonism of IL-18 reduces collagen deposition and enhances metalloproteinase activity in myeloid-specific NLRP3 gain-of-function mutant mice [106]. Additionally, extracellular ATP activates the inflammasome via the purinergic receptor P2RX7, linking it to immune responses that include NLRP3 activation and IL-1β release [107].

Recent findings emphasize that lipid metabolism significantly influences inflammasome activation in MASH. Peroxisome proliferator-activated receptors (PPARs) are critical regulators of liver and adipose metabolism. The gene Perilipin 5 (PLIN5), regulated by PPAR-α and implicated in lipid peroxidation, was shown to mediate inflammasome activation [108]. PLIN5-deficient mice on a high-fat diet demonstrated reduced lipid accumulation and lower inflammasome signaling. Comprehensive PPAR activation using experimental agents more effectively reduced liver inflammation and fibrosis than single PPAR agonists.

Sphingolipids, key components of cellular membranes, also play important roles in inflammation and metabolism. Sphingomyelin synthetase 1, which is overexpressed due to metabolic liver injury, drives pyroptosis in hepatic cells via the NLRC-4 inflammasome pathway. Additionally, sphingosine-1-phosphate (S1P) and its receptor S1PR2 are implicated in NLRP3 inflammasome activation, with S1PR2 silencing showing promising anti-inflammatory and antifibrotic effects [109]. S1PR4, which is consistently overexpressed in liver models, also participates in this process; inhibiting S1PR4 ameliorates hepatic inflammation and fibrosis by targeting the NLRP3 inflammasome. Recent studies have shown that sphingosine d18:1 can inhibit the interaction between HIF-2α and ARNT, thus suppressing the transcriptional activity of HIF-2α in hepatic macrophages, which in turn promotes NASH [110]. This suggests that macrophage HIF-2α may be a potential therapeutic target for treating NASH.

Lastly, chronic exposure to lipotoxic metabolites can induce endoplasmic reticulum (ER) stress and NLRP3 inflammasome activation, further contributing to liver inflammation and cell death [111]. ER stress mediated by transcription factors through the NLRP3 pathway exacerbates disease progression; thus, inhibiting the unfolded protein response may help alleviate these detrimental effects.

### 5.2. Pharmacological Treatment of MASLD/MASH

The management of MASLD and MASH is an evolving field, with recent advances in pharmacological therapies aimed at addressing the underlying metabolic dysfunction and hepatic inflammation. Current treatment strategies emphasize lifestyle modifications, including weight loss and physical activity, which have demonstrated significant benefits in reducing liver fat and inflammation. Nevertheless, pharmacotherapy is increasingly recognized as a vital component of treatment, particularly for patients who do not achieve adequate results through lifestyle changes alone.

Recent clinical trials have highlighted various agents under investigation, including glucagon-like peptide-1 (GLP-1) receptor agonists, sodium/glucose cotransporter-2 (SGLT-2) inhibitors, and peroxisome proliferator-activated receptor (PPAR) agonists [112,113]. These agents have demonstrated efficacy in reducing liver fat and improving liver histology. Advancements in understanding the inflammasome pathway have facilitated the development of therapeutics aimed at modulating inflammasome activation and signaling in MASLD. Noteworthy progress in both preclinical research and clinical trials in this area further underscores the potential of these therapeutic strategies.

Acetyl-CoA carboxylase inhibitors are primarily indicated for liver fibrosis and steatosis associated with non-alcoholic fatty liver disease (NAFLD). NASH is characterized by disrupted lipid metabolism in hepatocytes, resulting in increased de novo lipogenesis (DNL) and impaired fatty acid oxidation, which can lead to inflammation and fibrotic responses [114]. The regulation of DNL is crucial for fatty acid metabolism, with ACC being a key enzyme in this process. GS-0976, an allosteric ACC inhibitor, has shown promise in clinical trials. Phase I studies indicated a reduction in liver fat and fibrosis markers after 12 weeks in NASH patients, while phase II trials confirmed significant improvements in fibrosis and steatosis in individuals with advanced liver disease [115]. A recent phase IIb trial suggested that the combination of GS-0976 and cilofexor could effectively reduce fibrosis levels in patients with bridging fibrosis or compensated cirrhosis [116]. This combination, thus, represents a potential long-term treatment strategy for patients with advanced NASH-related liver fibrosis.

Caspase inhibitors, including pan-caspase inhibitors such as VX-166 and IDN-7314, have shown variable success in murine models of MASH [117,118]. Although emricasan failed to yield positive outcomes for decompensation or mortality when tested in patients with decompensated MASH cirrhosis, a selective caspase-1 inhibitor effectively reduced inflammatory responses and fibrosis in a transgenic MASH model [119]. However, the long-term inhibition of caspases raises concerns about potential hepatocarcinogenesis.

Gasdermin D inhibitors, such as necrosulfonamide, have reduced mortality in acute liver failure models [120]. In NLRP3-mutant mice, the inhibition of ASK1 alleviated liver fibrosis and inflammation [121]; however, phase III trials of the ASK1 inhibitor selonsertib did not demonstrate benefits in patients with MASH or severe alcoholic hepatitis (AH) compared to standard treatment [65].

NLRP3 (NOD-, LRR- and pyrin domain-containing protein 3) inhibitors such as the small molecule MCC950 have shown promise in preclinical models, improving liver injury and fibrosis. Despite positive effects in various animal studies, phase II trials in rheumatoid arthritis patients were halted due to concerns about liver toxicity [122]. Another NLRP3 inhibitor, CY-09, demonstrated improvements in obesity and insulin resistance in models of MAFLD, warranting further exploration in the context of MASH [123,124].

While therapies targeting inflammasome pathways show potential in managing liver disease, careful design and standardization of clinical trials are essential to achieving optimal results. Combination therapies, such as pemafibrate and dapagliflozin, are also being explored for their synergistic effects on lipid metabolism and glycemic control [113].

CCR2/CCR5 antagonists, such as cenicriviroc, are designed to combat liver fibrosis associated with conditions such as NASH through their anti-inflammatory and antifibrotic effects. Clinical trials have shown that cenicriviroc can significantly lower liver fibrosis markers over two years of treatment in NASH patients, although it does not address the fatty liver component of the disease [125]. Improvements in systemic inflammatory markers were also observed. In trials focused on primary biliary cholangitis, cenicriviroc reduced serum alkaline phosphatase levels while maintaining a favorable safety profile. Additionally, cenicriviroc shows potential in improving liver fibrosis in HIV patients while effectively suppressing the virus, demonstrating benefits across multiple fibrosis-related indices [126].

Research has shown that herbs may have potential therapeutic value for metabolic-associated steatotic hepatitis (MASH), such as ethanol extracts of *Artemisia argyi* (AA). The AA ethanol extract exhibits therapeutic effects on CDAHFD-induced liver disease by regulating NASH-/fibrosis-related factors and the composition of the gut microbiota. Notably, AA treatment reduced the abundance of potential pro-fibrotic bacteria (e.g., *A. ethanolgignens*) [127]. These findings suggest that AA is a promising candidate for the treatment of NASH-induced fibrosis.

Probiotics refer to live microorganisms that are beneficial to the host’s health, commonly including certain lactic acid bacteria (such as Lactobacillus and Bifidobacterium). Probiotics can reduce liver inflammation by lowering inflammatory markers (such as TNF-α and IL-6), thereby alleviating the inflammatory response in the liver. Additionally, probiotics can ferment dietary fibers to produce short-chain fatty acids (SCFAs) such as butyrate, acetate, and propionate. SCFAs have a protective effect on the liver, improving metabolic abnormalities and reducing liver fat accumulation, which in turn slows the progression of liver fibrosis.

Resmetirom is a “first-in-class”, once-daily oral thyroid hormone receptor (THR)-β-selective agonist designed to target the key underlying causes of NASH. Thyroid hormones play a central role in liver function by activating β receptors in liver cells, influencing a range of health parameters, from serum cholesterol and triglyceride levels to pathological fat accumulation in the liver. Resmetirom is highly selective, avoiding the activation of THR-α receptors outside the liver (including in the heart and skeletal muscles), and is specifically taken up in the liver. Previous clinical trials have demonstrated its safety, with no activity on THR-α receptors, no impact on skeletal or cardiac parameters, and no interference with other thyroid hormone pathways [128,129,130]. This therapy has been granted breakthrough therapy designation by the US FDA for NASH treatment.

Lanifibranor is a small-molecule PPAR agonist. Its advantage lies in its ability to moderately activate three PPAR isoforms, achieving the balanced activation of PPARα and PPARδ and the partial activation of PPARɣ. This induces antifibrotic, anti-inflammatory, and beneficial effects on human metabolism. It is the only pan-PPAR agonist currently in clinical development. Activating the three PPAR isoforms regulates metabolic levels, steatosis, inflammation, and fibrosis in NASH patients. Additionally, lanifibranor has shown significant benefits in preclinical models of decompensated cirrhosis, leading to marked improvements in fibrosis and portal hypertension. In the previously announced phase IIb NATIVE study, lanifibranor met the primary and key secondary endpoints, including NASH resolution without worsening fibrosis and improvement in liver fibrosis without worsening NASH. Additionally, this study is the first clinical trial to demonstrate efficacy on composite histological endpoints for NASH resolution and fibrosis improvement [131].

Moreover, the identification of biomarkers for the early detection and monitoring of disease progression is crucial for tailoring treatment strategies, as highlighted by ongoing research into non-invasive diagnostic tools [132,133]. Overall, the future of MASLD and MASH management lies in a personalized approach that integrates pharmacotherapy with lifestyle interventions, addressing both hepatic and extrahepatic manifestations of metabolic dysfunction. All the drugs or compounds mentioned in this part can be found in Table 2.

### 5.3. Alcoholic Liver Disease (ALD) and Liver Fibrosis

Alcoholic liver disease (ALD) is liver damage caused by long-term alcohol consumption, including conditions such as fatty liver, alcoholic hepatitis, and alcoholic liver fibrosis. Globally, ALD has become one of the major causes of liver disease. It is commonly observed in middle-aged individuals, typically between the ages of 30 and 60. As alcohol consumption has increased, the incidence of ALD among younger populations has also risen. ALD often coexists with other diseases, such as obesity, type 2 diabetes, metabolic syndrome, and other liver diseases (e.g., viral hepatitis), and these comorbidities can exacerbate the progression of ALD. The development of ALD can lead to liver fibrosis and, in severe cases, cirrhosis.

The pathogenesis of ALD involves complex interactions among several factors, including oxidative stress, inflammation, and gut microbiota dysbiosis. Ethanol metabolism in the liver produces acetaldehyde, a toxic metabolite that induces hepatocyte injury and promotes inflammation through the activation of inflammatory pathways such as the nuclear factor kappa B (NF-κB) pathway [134]. Furthermore, alcohol consumption alters the composition of gut microbiota, leading to increased intestinal permeability and the translocation of bacteria and their products into the liver, which exacerbates liver injury [135]. Mitochondrial dysfunction has also been recognized as a critical mechanism in ALD, leading to impaired energy metabolism and increased apoptosis of liver cells [136]. Recent studies have emphasized the role of SIRT1, a NAD+-dependent deacetylase, in mediating protective effects against alcohol-induced liver injury, suggesting that modulating this pathway could offer therapeutic benefits [137].

Research indicates that alcohol consumption triggers pro-inflammatory responses in liver cells, particularly through the activation of the NLRP3 inflammasome and caspase-1 (CASP-1), leading to the release of IL-1β in ethanol-fed mouse models [138]. Notably, there is a significant increase in the expression of inflammasome components in hepatic immune cells compared to parenchymal cells, with knockout models for NLRP3, CASP-1, or ASC lacking these inflammatory processes. The activation of the NLRP3 inflammasome is further influenced by hepatocyte-derived signals; uric acid and ATP released during alcohol-related liver injury act as secondary stimuli that enhance IL-1β processing and release, indicating a complex interplay between hepatocytes and hepatic immune cells [139]. Kupffer cells (KCs) have been implicated as key players, exacerbating inflammasome activation and promoting the recruitment of natural killer (NK) cells [140].

Efforts to mitigate the inflammatory effects of alcohol have encountered challenges. Trials using TNF-α antibodies were halted due to an increased risk of infections, whereas the use of an IL-1 receptor antagonist (IL-1Ra) in ethanol-fed mice resulted in reduced liver damage and improved survival compared to saline-treated controls. A recent investigation into the NLRP6 inflammasome revealed its role in modifying intestinal health during alcohol-induced liver disease. NLRP6-deficient mice exhibited alterations in the colonic epithelium and microbiota but did not show increased liver injury severity, although hepatic immune cell recruitment was significantly reduced. Continued alcohol exposure initiates a cascade of hepatic events, including increased CYP2E1 levels, excessive reactive oxygen species (ROS) production, and TLR4/MyD88/NF-κB pathway activation, promoting oxidative stress and liver damage [141,142,143]. Moreover, thioredoxin-interacting protein (TXNIP) plays a critical role in the intracellular activation of the NLRP3 inflammasome [144]. Ethanol exposure leads to elevated TXNIP expression in both animal models and liver biopsies from patients with alcoholic steatosis hepatitis (ASH) [145]. A key regulatory mechanism involves spleen tyrosine kinase (SYK), which promotes the phosphorylation of ASC and facilitates NLRP3 assembly. Elevated SYK levels in the livers of ethanol-fed mice correlate with the increased activation of macrophages and the production of pro-inflammatory cytokines [146]. Inhibiting SYK in animal models resulted in decreased inflammation and liver injury, while knocking out the P2RX7 gene, which is crucial for ATP-mediated NLRP3 activation, led to lower IL-1β levels and improved liver histology in ethanol-fed mice [147]. Additionally, transgenic mice expressing uricase, which metabolizes uric acid, displayed significant protection against ethanol-induced liver damage. Collectively, these findings underscore the complex mechanisms by which inflammasome pathways contribute to the pathogenesis of alcohol-related liver diseases and highlight potential therapeutic targets within these pathways.

### 5.4. Pharmacological Treatment of ALD

Current pharmacological strategies for managing ALD focus on reducing alcohol consumption, alleviating liver inflammation, and preventing the progression of fibrosis. Corticosteroids, such as prednisolone, are commonly used for patients with severe alcoholic hepatitis to mitigate inflammation and improve short-term survival [134]. Additionally, emerging agents targeting specific pathways involved in the pathogenesis of ALD are under investigation. For instance, the NLRP3 inflammasome has been recognized as a promising therapeutic target, with studies indicating that its inhibition may reduce liver inflammation and fibrosis in ALD models [148]. Furthermore, micronutrient supplementation has been explored for its potential role in modulating the inflammatory response and improving liver function [149]. Recent clinical trials have also focused on microbiome-based metabolic therapeutic approaches, aiming to restore gut microbiota balance and enhance liver health in ALD patients [150].

Anakinra, an IL-1 receptor antagonist approved for rheumatoid arthritis, has shown promise in murine models of ethanol-induced liver injury by improving hepatic parameters and survival rates [151]. However, a randomized controlled trial (RCT) combining Anakinra with zinc and pentoxifylline for severe alcoholic hepatitis did not show significant improvements in survival compared to treatment with prednisolone, although some survival benefits were noted at three and six months [152]. Another ongoing RCT is examining the efficacy of canakinumab, a human monoclonal antibody against IL-1β, in patients with alcoholic hepatitis.

In summary, continued clinical research is crucial for identifying effective treatment strategies that can improve the outcomes of individuals suffering from ALD and its associated complications. All the drugs or compounds mentioned in this part can be found in Table 2.

### 5.5. Viral Hepatitis and Liver Fibrosis

Viral hepatitis includes several types, primarily hepatitis A, B, C, D, and E, each with unique pathophysiological mechanisms and implications for liver fibrosis. Among them, hepatitis B virus (HBV) and hepatitis C virus (HCV) are particularly notorious due to their association with chronic liver disease and the progression of fibrosis. Chronic HBV infection can result in significant liver inflammation and fibrosis, with the degree of fibrosis often correlating with the duration of infection and the viral load. For instance, in patients with chronic hepatitis B, the presence of HBeAg is linked to higher levels of liver fibrosis compared to HBeAg-negative individuals, underscoring the role of viral replication in fibrogenesis [153].

Similarly, HCV infection is a major contributor to liver fibrosis, with research suggesting that the duration of infection and comorbidities such as obesity and diabetes can exacerbate fibrotic changes [154]. Additionally, the interaction between the virus and the host immune response is critical in fibrosis progression; chronic inflammation can activate hepatic stellate cells, which are the primary effector cells in liver fibrogenesis [155]. The situation is further complicated by the emergence of new viral strains, such as the Hepatitis Delta Virus (HDV), which co-infects with HBV and often results in accelerated liver damage and fibrosis [156]. Understanding the specific type of viral hepatitis and its underlying mechanisms is essential for developing targeted therapeutic strategies to mitigate liver fibrosis.

The relationship between viral clearance and the reversal of liver fibrosis has become a significant focus of recent research. Achieving a sustained virologic response (SVR) in patients with chronic hepatitis C through direct-acting antivirals (DAAs) has been associated with considerable reductions in liver stiffness and fibrosis scores [157]. Long-term studies indicate that patients who attain SVRs may experience fibrosis regression, with some even reverting to normal liver histology over time [158].

In contrast, for chronic hepatitis B, while antiviral therapy can effectively suppress viral replication, the complete reversal of liver fibrosis is less predictable. This outcome may depend on multiple factors, including the extent of baseline fibrosis and the duration of antiviral treatment [159]. Recent findings suggest that the timing of viral clearance is crucial; early intervention in disease progression is associated with better outcomes regarding fibrosis regression. Additionally, the presence of metabolic syndrome may complicate the relationship between viral clearance and fibrosis, potentially hindering liver tissue regeneration and promoting ongoing fibrogenesis, even after successful antiviral treatment [160]. Although achieving viral clearance is a critical goal in managing viral hepatitis, it is equally important to consider the broader context of liver health and metabolic factors when assessing fibrosis progression and potential reversal.

### 5.6. Antiviral Drug Therapy

Recent advancements in antiviral drug therapy have significantly transformed the management of viral hepatitis and its associated complications, particularly liver fibrosis. The introduction of direct-acting antivirals (DAAs) for hepatitis C has revolutionized treatment paradigms, offering high cure rates with minimal side effects [161]. DAAs work by targeting specific stages of the viral life cycle, leading to rapid viral clearance and subsequent improvements in liver histology. Clinical trials have revealed that patients treated with DAAs can achieve SVR rates exceeding 95%, along with measured reductions in liver stiffness via elastography [14].

In hepatitis B management, new oral antiviral agents, including nucleos(t)ide analogs, have also shown promise in suppressing viral replication and reducing liver inflammation, which can subsequently influence fibrosis progression. Ongoing research is exploring combination therapies that integrate immune-modulating agents with antiviral drugs to improve treatment efficacy and promote liver regeneration [162]. Despite these advances, a significant challenge remains in the long-term management of patients who have advanced liver disease, as these individuals continue to face an increased risk of hepatocellular carcinoma even after successful antiviral therapy [163]. Overall, the landscape of antiviral therapy is evolving rapidly, with continuous research focused on improving treatment outcomes for patients with viral hepatitis and related liver fibrosis. All the drugs or compounds mentioned in this part can be found in Table 2.

### 5.7. Drug-Induced Liver Injury and Liver Fibrosis

Drug-induced liver injury (DILI) represents a significant cause of acute liver failure, characterized by a spectrum of pathological changes, including hepatocyte necrosis, inflammation, and fibrosis. The molecular mechanisms underlying DILI are intricate, involving oxidative stress, mitochondrial dysfunction, and the activation of inflammatory pathways. For instance, an acetaminophen overdose can produce reactive metabolites that deplete glutathione, subsequently inducing oxidative stress and leading to hepatocyte death.

The activation of hepatic stellate cells (HSCs) is pivotal in the transition from liver injury to fibrosis. HSCs can be activated by various factors, such as transforming growth factor-beta (TGF-β), which promotes fibrogenesis and collagen deposition [164]. Additionally, the role of the NLRP3 inflammasome in DILI has gained attention, as it mediates inflammatory responses that exacerbate liver injury [165]. The interplay between oxidative stress and inflammation is central to the pathogenesis of liver fibrosis; persistent inflammation can activate fibrogenic pathways.

Furthermore, specific drugs, such as methotrexate, have been shown to induce hepatotoxicity through distinct molecular mechanisms, including the disruption of cellular homeostasis and the induction of apoptosis [166]. This multifaceted nature of DILI underscores the necessity of a comprehensive understanding of its underlying mechanisms to develop effective therapeutic strategies.

### 5.8. Treatment of Drug-Induced Hepatitis

The management of drug-induced hepatitis has significantly evolved, focusing on the early recognition and withdrawal of the offending agent. Recent advances in our understanding of DILI pathophysiology have spurred the exploration of various therapeutic options aimed at mitigating liver damage. For instance, antioxidants have been investigated as a means to combat the oxidative stress associated with DILI. Compounds such as indole-3-carboxaldehyde have demonstrated promise in alleviating acetaminophen-induced liver injury by inhibiting both oxidative stress and apoptosis [167].

Additionally, there is increasing interest in herbal compounds, with studies suggesting that certain phytochemicals can modulate metabolic pathways to protect liver cells from hepatotoxic effects [168]. Novel therapeutic targets, including syndecan-1, have also been identified as potential biomarkers and therapeutic agents in liver diseases, including DILI [169].

Overall, the treatment landscape for drug-induced hepatitis is transitioning toward a more personalized approach, integrating both pharmacological and non-pharmacological strategies to enhance liver recovery and prevent progression to fibrosis. Ongoing research is crucial for elucidating the efficacy of these interventions and formulating guidelines for effectively managing DILI. All the drugs or compounds mentioned in this part can be found in Table 2.

### 5.9. Autoimmune Liver Disease and Liver Fibrosis

Autoimmune liver diseases are a group of liver disorders caused by autoimmune mechanisms, characterized by the immune system mistakenly attacking the body’s own liver tissue, leading to inflammation and liver damage [170]. This includes autoimmune hepatitis, primary biliary cholangitis, and primary sclerosing cholangitis [171]. The common feature of these diseases is a chronic inflammatory and fibrotic process in the liver, and these pathological changes can eventually lead to liver failure and cirrhosis. The symptoms of autoimmune liver disease may be related to the extent of liver damage. Patients often present with symptoms, such as fatigue, loss of appetite, nausea, and jaundice, while some may experience abdominal pain or discomfort in the liver area [172].

### 5.10. Treatment of Autoimmune Liver Disease

Traditionally, the management of AILD has relied on corticosteroids and azathioprine as first-line therapies. Immunosuppressive therapy plays a key role in various autoimmune diseases and organ transplantation. In recent years, researchers have conducted in-depth studies on the use of immunosuppressants, finding that they not only effectively control disease progression but also alleviate patient symptoms. For example, immunosuppressants such as cyclophosphamide and corticosteroids have been widely used in clinical practice for patients with central nervous system autoimmune diseases. These medications work by suppressing the immune response and reducing inflammation, thus relieving symptoms and improving the quality of life for patients. However, immunosuppressive therapy also carries certain risks of side effects, such as an increased risk of infection and malignancy. Therefore, when using immunosuppressants, doctors need to make individualized adjustments based on the specific condition of the patient, ensuring the safety and effectiveness of the treatment. However, recent studies have demonstrated the potential benefits of alternative immunosuppressive agents, such as mycophenolate mofetil and tacrolimus, particularly in patients who are intolerant to standard treatments or exhibit an insufficient response [173]. Furthermore, the efficacy of biologics, including anti-TNF agents and monoclonal antibodies targeting specific immune pathways, is being investigated for refractory cases of AIH [174]. Novel drugs such as obeticholic acid (OCA) have demonstrated promising antifibrotic effects in phase III clinical trials in patients with primary biliary cholangitis (PBC). Cilofexor (GS-9674) has also been confirmed to be effective for primary sclerosing cholangitis (PSC) in phase II clinical trials, while Tropifexor (LJN452) is currently undergoing phase I clinical studies and has shown good tolerance.

Rituximab is a CD20-targeting monoclonal antibody primarily used to treat certain types of lymphoma, leukemia, and autoimmune diseases. In recent years, its application in autoimmune hepatitis (AIH) has garnered increasing attention. Clinical studies have shown that rituximab can effectively treat AIH patients who do not respond well to conventional immunosuppressants, such as steroids and azathioprine. Research indicates that patients treated with rituximab show significant improvements in liver inflammation markers, liver function, and symptoms (“Rituximab is a safe and effective alternative treatment for patients with autoimmune hepatitis: Results from the ColHai registry”). Although initial study results are encouraging, further research is needed to assess its long-term efficacy and establish usage protocols. When using rituximab to treat autoimmune hepatitis, physicians should closely monitor patients for their responses and side effects.

The role of epigenetics in AILD is gaining attention, with research indicating that epigenetic modifications may influence both disease susceptibility and progression [175]. Understanding these mechanisms could facilitate the identification of novel therapeutic targets and biomarkers for personalized treatment strategies. Additionally, exploring stem cell therapies offers a promising avenue for future research, potentially providing regenerative options for liver damage associated with AILD [174]. All the drugs or compounds mentioned in this part can be found in Table 2.

**Table 2:** Different drugs for liver inflammation and the latest research.

Types of Liver Inflammation	Drug Name	Target	Latest Research	References
MASLD/MASH	Acetyl-CoA carboxylase inhibitors	Acetyl-CoA carboxylase	NCT04395950	[128,129,130,131,176,177,178,179,180,181]
	Caspase inhibitors (VX-166, IDN-7314) *	Caspase	-	
	Gasdermin D inhibitors	HSP47 siRNA	NCT06546059	
	NLRP3 inhibitors (MCC950)	NLRP3	ChiCTR2400091186	
	CCR2/CCR5 antagonists	CCR2/CCR5	NCT03767582	
	Probiotics, SCFAs	TNF-α, IL-6	ChiCTR2400091708	
	Resmetirom (C17H12Cl2N6O4, MGL-3196)	THR-β	CTIS2024-510627-20-00	
	Lanifibranor(C19H15ClN2O4S2)	PPAR	CTIS2023-508248-23-00	
Alcoholic Liver Disease	Corticosteroids (C_21_H_30_O_4_)	Factor of inflammation	ISRCTN17100156	[182,183,184,185,186,187]
	Anakinra (C_759_H_1186_N_208_O_232_S_10_)	IL-1 receptor	NCT06666335	
Viral Hepatitis	DAAs	Hepatitis C	NCT06658782	[188,189]
	Nucleos(t)ide analogs	Hepatitis B	NCT06668727	
Drug-Induced Liver Injury	Indole-3-carboxaldehyde(C_9_H_7_NO) *	Acetaminophen	-	[190,191,192,193]
	Syndecan-1	-	NCT06670248	
Autoimmune liver disease	OCA	FXR	NCT04971785	[194,195,196,197,198,199]
	Cilofexor (C_28_H_22_Cl_3_N_3_O_5_, GS-9674)	-	NCT04971785	
	Tropifexor (C_29_H_25_F_4_N_3_O_5_S, LJN452)	-	CTRI/2020/08/027184	
	Rituximab (C_6416_H_9874_N_1688_O_1987_S_44_)	Monoclonal antibody	NCT06664411	

* There are no relevant clinical studies.

## 6. Summary and Prospects

In conclusion, the intricate relationship between liver fibrosis and various forms of hepatitis underscores the complexity of hepatic pathophysiology. Liver fibrosis, which entails a progressive scarring process, primarily arises from chronic inflammation and injury, commonly seen in conditions, such as viral hepatitis, alcohol-related liver disease, metabolic dysfunction-associated steatotic liver disease (MASLD), drug-induced liver injury, and autoimmune hepatitis (AIH). Understanding the underlying molecular mechanisms is essential, as they not only elucidate the pathogenesis of liver fibrosis but also reveal potential therapeutic targets [200]. For example, signaling pathways involving transforming growth factor-beta (TGF-β) and platelet-derived growth factor (PDGF) are critical for the activation of hepatic stellate cells, which are pivotal in fibrosis development [201,202].

Pharmacological interventions show considerable promise in managing liver fibrosis. Current treatment strategies focus on halting or reversing the fibrotic process, with emerging therapies, including antifibrotic agents and immune modulators, demonstrating encouraging results in clinical settings. However, challenges persist in the therapeutic landscape, such as drug efficacy, patient compliance, and the need for individualized treatment approaches. It is vital to consider the findings of various studies, as some interventions may show promise in specific patient cohorts while yielding variable outcomes in others [64]. This heterogeneity demands nuanced interpretations of data and a comprehensive approach to clinical trial design.

The future of liver fibrosis research holds great promise, with a multifaceted approach that encompasses molecular biology, biomarker discovery, and the development of novel therapeutics. As we delve deeper into the molecular pathways that govern liver fibrosis, we are not only gaining a better understanding of the disease but also identifying potential targets for intervention. This is crucial for the development of personalized medicine, where treatments can be tailored to individual patients based on their unique genetic and molecular profiles [203].

The quest for biomarkers is particularly exciting, as these molecular indicators can predict treatment responses and disease progression. By identifying such biomarkers, we can stratify patients into groups that are most likely to benefit from specific therapies, thereby improving treatment efficacy and reducing unnecessary side effects [204,205]. This is especially important in conditions such as liver fibrosis, where the progression can be variable and unpredictable. Addressing the root causes of liver fibrosis, such as viral hepatitis, alcohol-related liver damage, and metabolic disorders, is paramount. This targeted approach can help mitigate the progression of the disease and, in some cases, even reverse the fibrosis. The combination of antiviral therapies with novel antifibrotic agents presents a promising frontier in the treatment landscape, potentially offering synergistic effects that enhance patient outcomes. The advent of organ-on-a-chip and liver organoid technologies is revolutionizing drug screening and preclinical testing [19]. These innovative platforms allow for more accurate modeling of human tissue responses, which can lead to the identification of more effective drugs with fewer side effects. By mimicking the complexity of the human liver, these technologies can provide a more nuanced understanding of drug interactions and efficacy, ultimately accelerating the drug development process [206,207,208]. The integration of advanced technologies, such as genomics and proteomics, is further unveiling new therapeutic targets and deepening our comprehension of liver disease progression. These technologies enable the analysis of the entire genome or the complete set of proteins expressed under specific conditions, providing insights into the complex interplay of genes and proteins that contribute to liver fibrosis [209,210]. This knowledge can lead to the development of targeted therapies that disrupt the disease process at its core. By fostering collaboration between basic researchers and clinical practitioners, we can ensure that scientific discoveries are translated into clinical practice, leading to more effective strategies to combat liver fibrosis. This collaborative approach is essential for bridging the gap between laboratory research and patient care, ensuring that new treatments reach those in need.

Overall, the future of pharmacotherapy for liver fibrosis and hepatitis looks optimistic. Ongoing research and technological advancements are shaping our approach to these complex conditions, offering hope for improved treatment options and better patient outcomes. As we continue to unravel the mysteries of liver fibrosis, we move closer to a future where this debilitating condition can be effectively managed and, potentially, cured.

## List of Abbreviations

Abbreviations	Full name
ECM	Extracellular matrix
HSCs	Hepatic stellate cells
HSPs	Heat shock proteins
PPAR	Peroxisome proliferator-activated receptor
TGF-β	Transforming growth factor-beta
PDGF	Platelet-derived growth factor
JAK/STAT3	Janus Kinase/Signal Transducer and Activator of Transcription 3
IL	Interleukin
Wnt/β-catenin	Wnt/beta-catenin signaling pathway
Notch	Notch Signaling Pathway
Hippo	Hippo Signaling Pathway
HCC	Hepatocellular carcinoma
NASH	Non-alcoholic steatohepatitis
MAFLD	Metabolic Associated Fatty Liver Disease
MASH	Metabolic dysfunction-associated steatohepatitis
ASK1	Apoptosis signal-regulating kinase 1
BRD4	Bromodomain-Containing Protein 4
FXR	Farnesoid X Receptor
SGLT-2	Sodium/glucose cotransporter 2
GLP-1	Glucagon-like peptide-1
DAAs	Direct-acting antivirals
DILI	Drug-induced liver injury
AIH	Autoimmune hepatitis
PBC	Primary biliary cholangitis
PSC	Primary sclerosing cholangitis
